# Augmented reality-enhanced 3D navigation system for managing isolated Onodi sinusitis: advantages and insights

**DOI:** 10.1097/MS9.0000000000005228

**Published:** 2026-06-09

**Authors:** Bassel Hallak, Salim Bouayed, Salma Bouez

**Affiliations:** a Department of Otorhinolaryngology-Head and Neck Surgery, Sion Hospital, Sion, Switzerland; b Department of Anesthesiology, Sion Hospital, Sion, Switzerland

**Keywords:** 3D navigation system, augmented reality technology, case report, imaging, nasal endoscopy, Onodi sinusitis, surgical management

## Abstract

**Introduction and importance:**

The Onodi cell is a pneumatized posterior ethmoidal cell situated lateral and superior to the sphenoid sinus. It lies in close proximity to the posterior ethmoidal artery and the optic nerve. Isolated Onodi sinusitis is a rare condition but carries a high risk of optic nerve injuries.

**Presentation of case:**

We report the case of an 86-year-old Caucasian woman with unilateral isolated Onodi sinusitis, incidentally diagnosed on a routine CT scan performed to investigate a general decline in health. Diagnostic methods, appropriate management, and follow-up are described.

**Clinical discussion:**

The incidence of the sphenoethmoidal air cell, also known as the Onodi cell, has been reported to range from 51% to 60% in Asian cadaveric studies. Although isolated Onodi sinusitis is exceptionally rare, it has important clinical implications due to its close anatomical relationship with critical neurovascular structures. Early and accurate diagnosis, followed by appropriate management, is essential to ensure a favorable outcome.

**Conclusion:**

Isolated Onodi cell sinusitis is exceptionally rare. The management of this condition should be tailored to each individual clinical situation, combining surgical and medical approaches.

## Introduction

Pathological conditions affecting the paranasal sinuses are common, particularly acute and chronic sinusitis^[^1^]^. Owing to the close anatomical proximity between the paranasal sinuses and the orbits, inflammatory or infectious processes originating in the sinuses can easily extend to the orbital structures.

The intimate relationship between the Onodi cell and the optic nerve explains why inflammation within this cell can directly affect the optic nerve, which is highly sensitive to inflammatory damage.

Management of isolated Onodi sinusitis requires a tailored approach, with surgical drainage remaining the treatment of choice. A 3D imaging-guided navigation system-supported sinus surgery provides several advantages, particularly when combined with augmented reality (AR) technology.

We present the case of an elderly patient with multiple comorbidities who was incidentally diagnosed with unilateral isolated Onodi cell sinusitis during a routine CT scan. The patient was symptom-free. Clinical presentation, radiological and laboratory findings, medical and surgical management, outcomes, and follow-up are described.

HIGHLIGHTS

- Isolated Onodi cell sinusitis is exceptionally rare.
- In its early stages, this condition is often asymptomatic. Clinical signs and symptoms typically appear only in more advanced stages.
- The management of this condition should be tailored to each individual’s clinical situation, combining surgical and medical approaches.
- Image-guided navigation systems offer numerous advantages in sinus surgery. When combined with augmented reality technology, they provide even greater accuracy and precision, further enhancing surgical outcomes.

This case report has been prepared in line with the SCARE checklist^[^2^]^.

## Presentation of case

### Patient information

A woman in her 80s was admitted to the geriatrics department for investigation of a general decline in her health status. Her medical history included arterial hypertension, paroxysmal atrial fibrillation, a recent acute ischemic stroke in the left Sylvian artery territory, dyslipidemia, and overweight (BMI 29.02 kg/m^2^).

She was a former smoker who had quit 20 years earlier and reported no alcohol consumption. There was no history of recurrent sinusitis or prior nasal or sinus surgery. She was on multiple medications, including Eliquis 2.5 mg twice daily for anticoagulation therapy.

### Clinical findings

**Figure 1.**
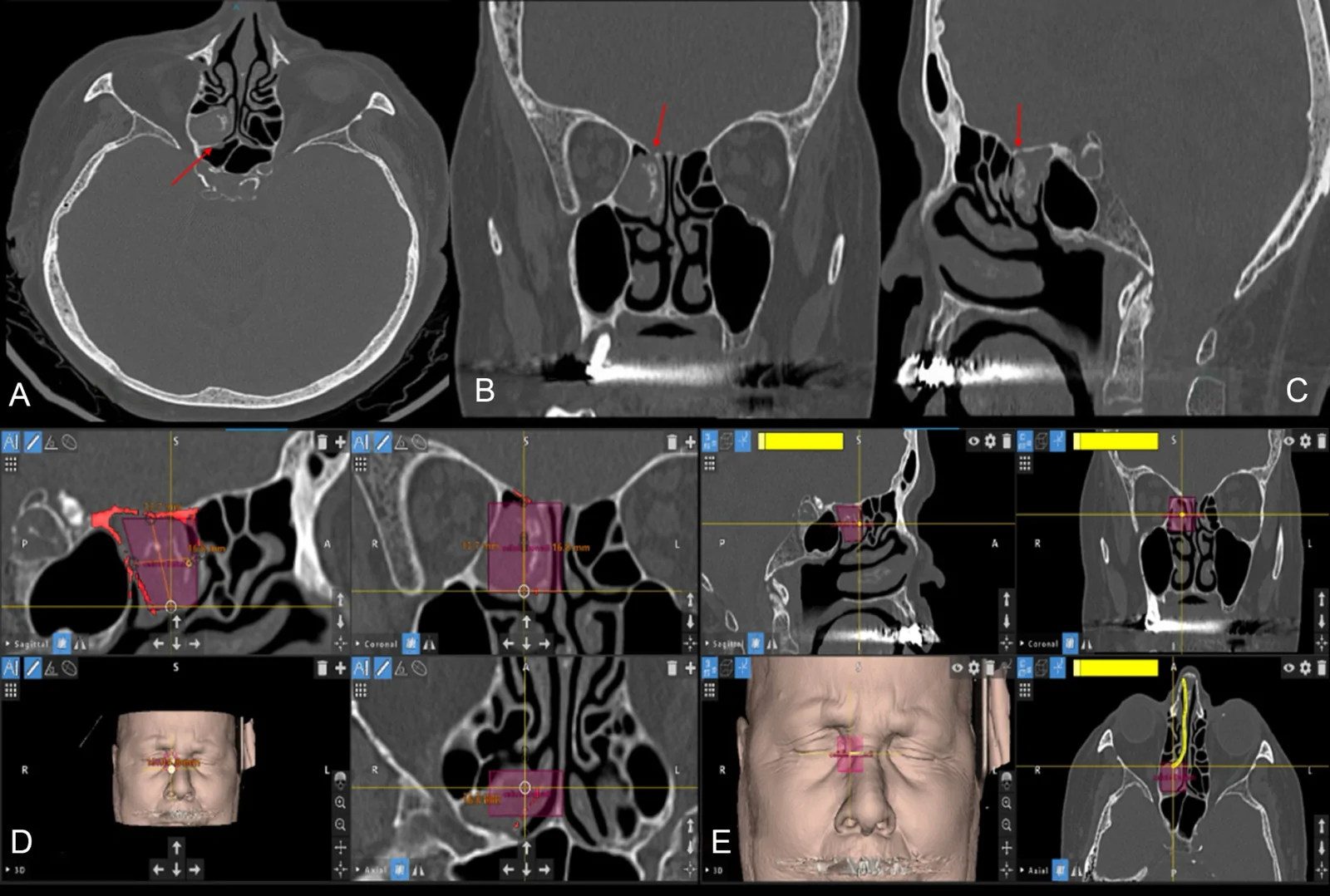
(a–c) Preoperative CT scan images (axial, coronal, and sagittal) showed total opacification of right Onodi cell with multiple calcifications and areas of bony lysis. (d and e) Preoperative 3D planification of right Onodi cell with AR navigation system.

CT imaging revealed complete opacification of the right Onodi cell with calcifications, signs of osteitis, and focal areas of bony lysis involving the sinus walls (Fig. 1a–c).

The remaining paranasal sinuses were well-pneumatized, and no orbital or intracranial complications were identified. These radiological findings were highly suggestive of fungal sinusitis. The patient remained asymptomatic.

Nasal fibroendoscopy revealed no abnormalities. The nasal mucosa appeared normal, with no purulent discharge, polyps, or intranasal lesions.

### Diagnostic assessment

#### Diagnostic reasoning

Given the patient’s advanced age, multiple comorbidities, and imaging findings suggestive of invasive fungal sinusitis, with bacterial infection or neoplastic involvement of the right Onodi cell also considered in the differential diagnosis, surgical sinus exploration was deemed necessary and subsequently performed. The patient accepted the management strategy.

### Therapeutic intervention

#### Pre-intervention considerations

Preoperative evaluation included complete blood tests, chest X-ray, and electrocardiography, all of which were unremarkable, along with an anesthesiologic assessment. Anticoagulation therapy was maintained because of the patient’s recent acute ischemic stroke. Surgery was performed under general anesthesia.

#### Intervention

The procedure was carried out via an endoscopic intranasal approach using a 3D image-guided navigation system combined with AR technology (Stryker Scopis ENT Navigation System with Target Guided Surgery, Stryker^®^, USA). Preoperative planning included a 3D reconstruction of the Onodi cell and precise measurements in all spatial planes. The surgical access pathway was carefully defined and integrated into the AR navigation system (Fig. 1d,e).

#### Peri-intervention details

**Figure 2.**
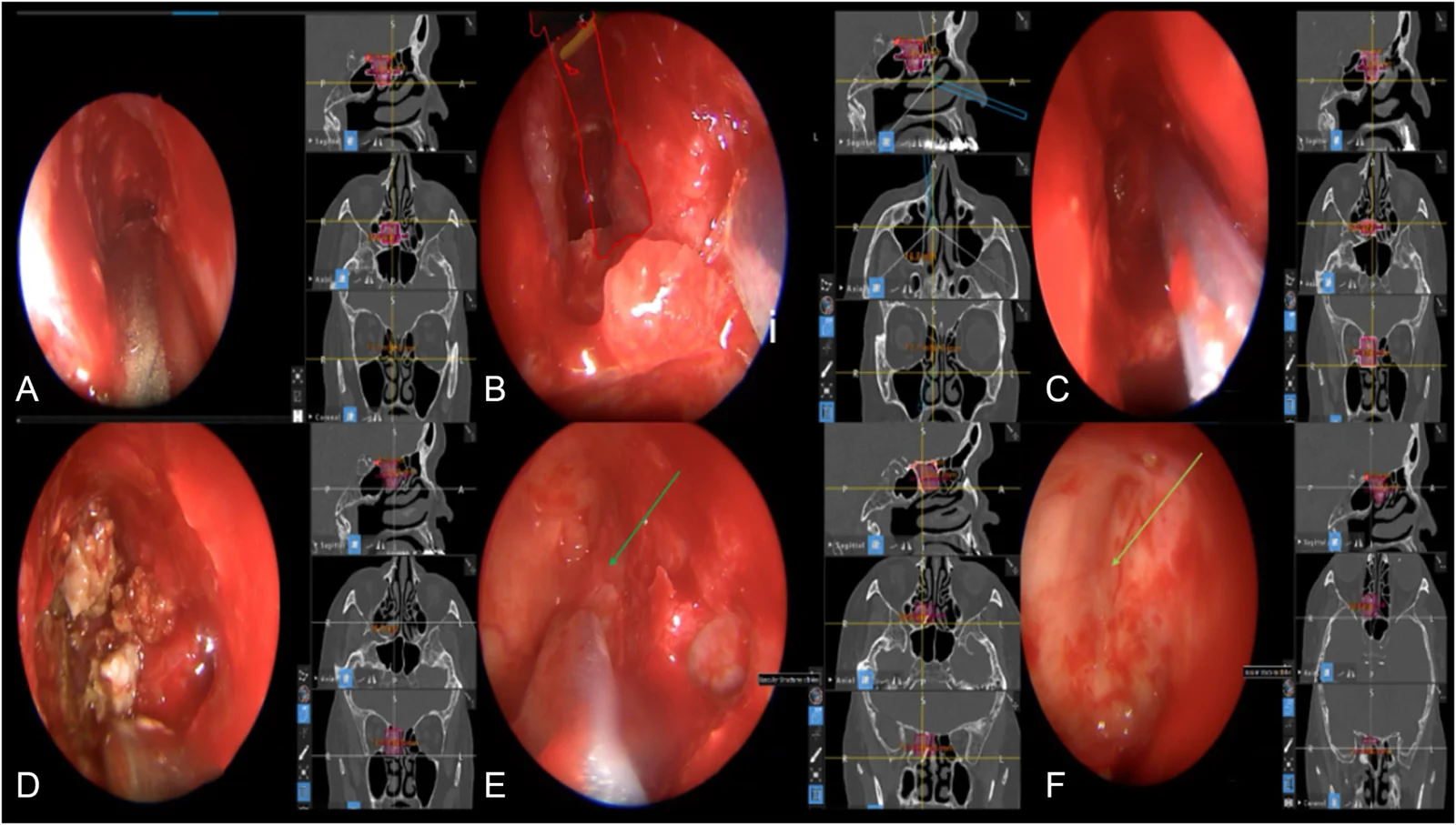
(a–d) Intraoperative endoscopic images showed direct access to the right Onodi cell guided with 3D navigation system combined with AR technology. (e and f) Intraoperative endoscopic images showed right Onodi cell after complete drainage with areas of bony lysis.

A rigid 0° nasal endoscope was used to identify various intranasal anatomical landmarks, and the direct pathway to the Onodi cell had been pre-planned in the navigation system. The target cell was approached considering its posterolateral location relative to the sphenoid sinus. Endoscopic exposure was performed in real time with dynamic 3D imaging (Fig. 2a–c). The Onodi cell was opened, revealing it completely filled with purulent secretions containing debris resembling fungal filaments (Fig. 2d). Complete drainage and evacuation of the Onodi cell were achieved, and multiple samples were collected for microbiological analysis.

Following complete drainage, endoscopic inspection revealed multiple areas of bony lysis, while the periosteum remained intact (Fig. 2e,f). Intraoperative bleeding was moderate and effectively controlled with gauze packing and bipolar cauterization. Hemostatic and absorbable materials were subsequently placed within the nasal cavity.

#### Post-intervention management

Postoperative management consisted of the intravenous administration of co-amoxicillin (1.2 g) twice daily for 7 days, appropriate analgesia, and local nasal care with daily irrigation using a physiological saline solution. The postoperative course was uneventful, with no bleeding or other complications observed.

Microbiological analysis of the samples revealed no growth of aerobic bacteria but showed a strong presence of anaerobic bacteria, with *Peptoniphilus* species (*Peptostreptococcus*) identified as the pathogenic agent. Surprisingly, the mycological examination was negative, with no fungal organisms detected. Based on these results, consultation with an infectious disease specialist was obtained, and the initial antibiotic regimen was considered adequate, with no additional therapy required. In the absence of neurological, ocular, or other concerning signs, additional specialist consultations were deemed unnecessary.

### Follow-up and outcomes

Follow-up assessments in outpatient consultations at 2, 4, and 6 months post-surgery confirmed a stable and favorable evolution, with no complications. Successive nasal endoscopic evaluations during the follow-up demonstrated satisfactory mucosal healing.

Considering the favorable clinical course and the absence of signs of recurrence or complications, no additional imaging studies were performed.

The patient tolerated the treatment well, with no complications or adverse events (Clavien-Dindo Grade 0).

## Discussion

Adolf Onodi first described the Onodi cell in 1904 as an anatomical variation of the most posterior ethmoidal air cell, which has been reported in 7–65% of people. Onodi cells remain asymptomatic unless affected by sinus disease^[^3^]^. Reported causes of Onodi cell sinusitis include bacterial or fungal infections, as well as inflammatory changes secondary to compressive lesions such as polyps, mucoceles, or even necrotic tumors^[^4^]^.

The reported incidence of Onodi cells identified on CT ranges from 8 to 30%, with a higher prevalence observed in Asian populations^[^5^]^. Characteristic features include their lateral and superior pneumatization relative to the sphenoid sinus, their close proximity to the optic canal, and the prominence of the optic nerve tubercle or the internal carotid artery^[^6^]^.

The pathophysiological mechanism underlying optic neuropathy secondary to Onodi cell sinusitis is likely multifactorial, involving both mechanical compression and inflammatory processes^[^7^]^.

Functional endoscopic sinus surgery is considered the gold standard for the surgical management of paranasal sinus diseases. Since the late 1980s, navigation systems have been used for image-guided sinus surgery^[^8^]^. Their main advantage lies in providing more accurate intraoperative anatomical orientation, thereby facilitating surgical guidance. In addition, they help reduce the surgeon’s mental workload. Navigation systems have undergone continuous improvements in both quality and accuracy, as well as in their software capabilities. Among these advancements is the integration of AR elements, which rely on the fundamental principle of superimposing preoperative imaging onto the conventional endoscopic view.

Few studies have investigated the benefit of AR in navigation sinus surgery. Li *et al* developed a system capable of fusing endoscopic images with 3D virtual images^[^9^]^. Leonard *et al* developed a video-based navigation system enabling a surgeon to asynchronously register a sequence of endoscopic images to a CT scan^[^10^]^.

Isolated Onodi sinusitis is a rare condition that has been reported in the literature mainly through sporadic case reports. In our case, the patient was entirely asymptomatic, and the diagnosis was made incidentally during a routine CT scan. Although the imaging findings were highly suggestive of a fungal infection, the final microbiological analysis demonstrated bacterial elements, underscoring the discrepancy between the radiological appearance and the actual pathology.

Given the patient’s multiple comorbidities and the impossibility of discontinuing anticoagulant therapy, minimizing operative time and limiting surgical maneuvers were essential. In this setting, the AR navigation system proved particularly valuable by providing highly precise and direct access to the target cell. To the best of our knowledge, no similar clinical case managed with the same technology and surgical approach has been described in the literature.

## Conclusion

Imaging findings may be subtle or nonspecific and do not always correlate with the underlying pathology. This limitation may be partly explained by the fact that different conditions can present with similar radiological features. Image-guided navigation systems combined with AR technology provide several advantages in sinus surgery, including improved accuracy and precision, more direct access to the target cell with fewer surgical manipulations, and a reduction in operative time. The main limitations of our report include its single-case design, the absence of a standardized consensus regarding management, and the limited availability and cost of this technology.

## Data Availability

The data are available and ready to use, if needed.

## Research registration unique identifying number (UIN)

No registration number for a single case report.

## Artificial intelligence

No artificial intelligence (AI) tools were used in the research, data analysis, or manuscript preparation. The case has been written in accordance with SCARE guidelines.
